# Energetic Glaucoma Segmentation and Classification Strategies Using Depth Optimized Machine Learning Strategies

**DOI:** 10.1155/2021/5709257

**Published:** 2021-11-25

**Authors:** V. Elizabeth Jesi, Shabnam Mohamed Aslam, G. Ramkumar, A. Sabarivani, A. K. Gnanasekar, Prince Thomas

**Affiliations:** ^1^Department of Networking and Communications, College of Engineering and Technology, SRM Institute of Science and Technology, Kattankulathur, Chennai, Tamilnadu, India; ^2^Department of Information Technologies, College of Computer and Information Sciences, Majmaah University, AlMajmaah 11952, Saudi Arabia; ^3^Department of Electronics and Communication Engineering, Saveetha School of Engineering, SIMATS, Chennai, Tamilnadu, India; ^4^Department of Electronics and Instrumentation Engineering, Sathyabama Insitute of Science and Technology, Chennai, Tamilnadu, India; ^5^Department of Electronics and Communication Engineering, Rajalakshmi Institute of Technology, Chennai, Tamilnadu, India; ^6^School of Computing, Woldia Institute of Technology, Woldia University, Woldia, Ethiopia

## Abstract

Glaucoma is a major threatening cause, in which it affects the optical nerve to lead to a permanent blindness to individuals. The major causes of Glaucoma are high pressure to eyes, family history, irregular sleeping habits, and so on. These kinds of causes lead to Glaucoma easily, and the effect of such disease leads to heavy damage to the internal optic nervous system and the affected person will get permanent blindness within few months. The major problem with this disease is that it is incurable; however, the affection stages can be reduced and the same level of effect as that for the long period can be maintained but this is possible only in the earlier stages of identification. This Glaucoma causes structural effect to the eye ball and it is complex to estimate the cause during regular diagnosis. In medical terms, the Cup to Disc Ratio (CDR) is minimized to the Glaucoma patients suddenly and leads to harmful damage to one's eye in severe manner. The general way to identify the Glaucoma is to take Optical Coherence Tomography (OCT) test, in which it captures the uncovered portion of eye ball (backside) and it is an efficient way to visualize diverse portions of eyes with optical nerve visibility shown clearly. The OCT images are mainly used to identify the diseases like Glaucoma with proper and robust accuracy levels. In this work, a new methodology is introduced to identify the Glaucoma in earlier stages, called Depth Optimized Machine Learning Strategy (DOMLS), in which it adapts the new optimization logic called Modified K-Means Optimization Logic (MkMOL) to provide best accuracy in results, and the proposed approach assures the accuracy level of more than 96.2% with least error rate of 0.002%. This paper focuses on the identification of early stage of Glaucoma and provides an efficient solution to people in case of effect by such disease using OCT images. The exact position pointed out is handled by using Region of Interest- (ROI-) based optical region selection, in which it is easy to point the optical cup (OC) and optical disc (OD). The proposed algorithm of DOMLS proves the accuracy levels in estimation of Glaucoma and the practical proofs are shown in the Result and Discussions section in a clear manner.

## 1. Introduction

Glaucoma is the calm killer of vision; it is caused due to intraocular eye pressure. The general cause of Glaucoma is reduced eye sight and slowly it leads to a permanent vision loss, in which it is considered to be the world's second place eye vision loss perspective and blindness [1]. The analysis of World Health Organization reports that the number of people suffering from such disease in 2022 will reach nearly 80 million people. In general, the optical nerves of eyes are sending the visualization signals to the brain and internally there are millions of other nerve regions and this harm effect may prompt primary disfigurement of optical disc, also called Optical-Nervous-Head, in which at last it causes vision misfortune. As Glaucoma has no side effects in starting period and vision misfortune caused by the sickness is irreversible, early identification followed by treatment to limit the indications is vital. In order to notice the eye ball structure using OCT, pictures are procured by means of various methods counting, Ophthalmoscope is utilized for the immediate assessment of optical nerve system, and backhanded assessment is done by means of microscopy features [2]. Glaucoma can be identified based on various kinds of assessments, specifically morphologic as well as nonmorphologic feature measurements. Measurement of optical disc, optical cup, and neuroretinal edge region by means of Inferior-Superior-Nasal-Temporal (ISNT) rule are determined through morphologic enabled component extraction procedures. Retinal Nerve Fiber Layer (RNFL), Peripapillary Atrophy (PPA), and blood vessels go under nonmorphological feature estimations [3]. Computerized shaded OCT image is an arising procedure in the discovery of glaucoma as it is financially savvy in contrast with other methods; that is, optical intelligence and Heidelberg Retinal Tomography (HRT), so, for the proposed analysis, OCT images are utilized to identify the optical discs and cups with respect to Glaucoma measurements. For the investigation of retinal pictures, optic circle location is an emerging portion [4–6].

### 1.1. Glaucoma Types and Specifications

The Glaucoma can be classified into four different types: Open-Angle Glaucoma, Angle-Closure Glaucoma, Secondary Glaucoma, and Childhood Glaucoma. All these types are harmful and cause severe damage to human eyes with respect to systematic causes and lead to permanent blind stages. The following summary illustrates the visualization of all stages in detail with proper specifications.

#### 1.1.1. Open-Angle Glaucoma

**Figure 1:**
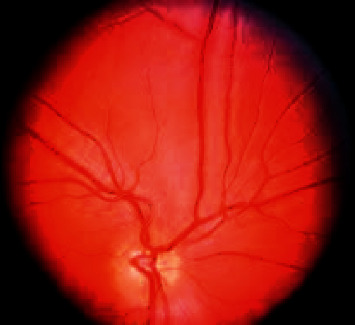
Open-Angle Glaucoma.

A general form of eye disease type is Open-Angle Glaucoma; it is a common type occurring over the cornea region of the eye ball, in which the IRIS state stays open. This will cause severe pressure to internal eye and eye stress suddenly, in which it leads to Optical Nerve Damage. This scenario leads an eye to a nonvisualized pattern in slow manner and it occurs so gradually that one may lose vision before even being mindful of an issue.  Figure 1 shows the modular view of Open-Angle Glaucoma, in which the level of effect is increased slowly and it causes serious effects to the inner region without any harmful symptoms [7, 8].

#### 1.1.2. Angle-Closure Glaucoma

**Figure 2:**
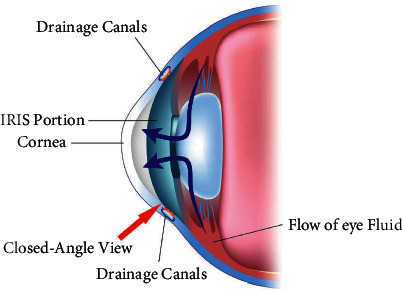
Angle-Closure Glaucoma.

The second form of Glaucoma is considered to be the angular form, also called Angle-Closure Glaucoma. This condition occurs when the cornea swells forward.  Figure 2 shows the modular view of Angle-Closure Glaucoma, in which the level of fluid in the inner region of eye is locked and the effect is serious and harmful in later stages [9].

Based on the view of the above figure, the visualization of Angle-Closure Glaucoma is clearly seen and, therefore, eye fluid cannot course through the eye and pressing factor increments. A few people have tight seepage points which put them at expanded danger of Angle-Closure Glaucoma. Angle-Closure Glaucoma may happen out of nowhere or steadily and acute closed-angle glaucoma is a health-related crisis [10].

#### 1.1.3. Secondary Glaucoma

**Figure 3:**
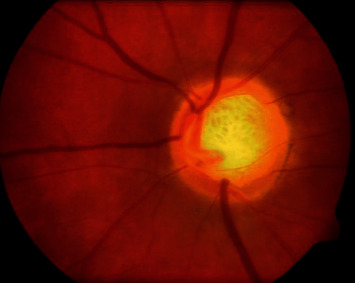
Secondary Glaucoma.

In typical strain glaucoma, the optical nerve becomes harmed despite the fact that one's eye pressure is inside the ordinary reach, and nobody knows the specific purpose behind this. It may have a delicate optical nerve or it may have less blood being provided to individual's optic nerve. This restricted blood stream could be brought about by atherosclerosis: the development of fat points in the supply routes or different conditions that debilitate blood flow. This condition of Glaucoma is called Secondary Glaucoma.  Figure 3 shows the modular view of Secondary Glaucoma.

#### 1.1.4. Childhood Glaucoma

**Figure 4:**
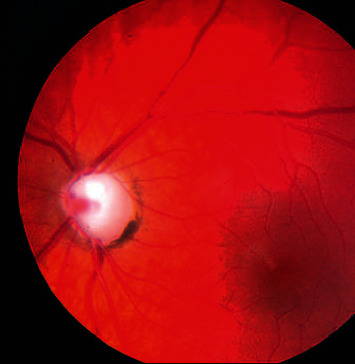
Childhood Glaucoma.

It is feasible for babies and kids to have Glaucoma in earlier stages and it very well might occur from birth or be created in the initial not many long periods of life. The optical nervous system harm might be brought about by waste blockages or a basic ailment on the eye region.  Figure 4 shows the modular view of Childhood Glaucoma.

### 1.2. Dataset Summary

**Figure 5:**
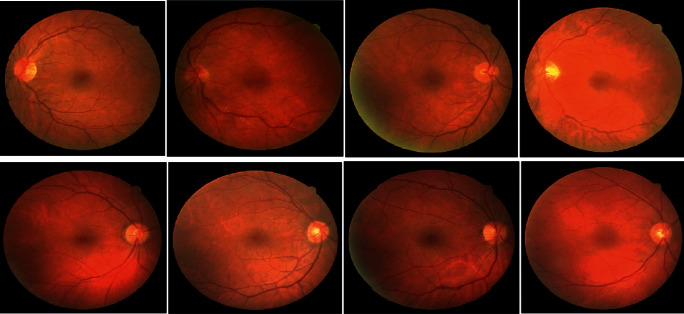
Different classes of glaucoma images available into the dataset.

This proposed approach adopts real-time modified Glaucoma disease dataset with the presence of multiple OCT image patterns with the association of several classes and the respective labels bind to the classes [11, 12]. Every class label indicates different types of Glaucoma disease combination and the Glaucoma constraint category can easily be identified with proper prediction principles.  Figure 5 shows the different views of OCT Glaucoma disease images with proper label indications accumulated from “OCT-Glaucoma disease dataset.” In this dataset, all the Glaucoma disease images are properly segmented without dominating background surroundings and the clarity of respective images is comparatively good with proper proportions of 256 × 256 pixel ranges. The Glaucoma disease dataset's segmentation process is systematic in terms of scripting and it provides good enough nature on associated dataset, and the proposed approach associates a new optimization logic in the summary during feature extraction stage of implementation for estimating the coloring range, brightness of the image, and the saturation key points of the Glaucoma disease image. One of the means of that handling too permitted us to effortlessly fix color casting, which turned out to be very solid in a portion of the subsets of the dataset, accordingly eliminating another possible predisposition [13, 14]. This arrangement of tests was intended to comprehend if the machine learning procedures really learn the idea of eye sicknesses or in the event that it is simply learning the intrinsic predispositions in the dataset.  Figure 5 shows the various renditions of a different Glaucoma disease dataset images view for an arbitrarily chosen set of disease types.

The remainder of this paper is organized as follows: related studies are in Section 2; Section 3 illustrates the proposed system methodologies in detail with proper algorithm flow and Section 4 illustrates the result and discussion portion of the paper, and Section 5 gives the conclusion and future scope of the proposed paper. These all will be explained in detail over the further section summaries.

## 2. Related Works

Wang et al. proposed a paper related to Glaucoma analysis based on joint retinal segmenting and classifying procedures. In that paper [15], the authors illustrated that identification of Glaucoma in the earlier stage provides a lot of benefits and saves human vision in clear manner. This system follows proposed flow with Optical Coherence Tomography (OCT) images, in which it is a well-known digital image format for identifying retinal diseases. The major objective of this work is to analyze the ophthalmologists, in which it provides the best treatment for the Glaucoma disease in an effective manner, but the logic of diagnosis is important for providing an effective treatment accordingly. So, a new model is proposed in this paper, called Bidecision Strategy Logic; it is designed with respect to deep learning principles. In this paper, the screening process of images is done through the analysis of cup to disc ratio and the resulting determination provides a good accuracy ratio of identifying the disease in outcome. At last, the classification principles take the retinal nerve fiber layer thickness vector as information and yield the likelihood of being Glaucoma. In the classification procedure, a painstakingly planned module is proposed to actualize the clinical procedure to analyze Glaucoma. This strategy is approved both in a gathered dataset ratio of 1004 OCT-B Scan analysis from 234 intense things and in a public dataset of 110 B Scans from 10 patients with diabetes-macular swelling. The major advantages identified from this paper are the application of the new decision-based algorithm called Bidecision Strategy Logic, and the accuracy level of Glaucoma disease prediction is high.

Fathima and Subhija proposed a paper related to Glaucoma identification with respect to fundus and Optical Coherence Tomography images. In this paper, the authors illustrated that Glaucoma is the major cause and leads to destroying one's vision within a small time period, and this kind of effect causes severe damage to the internal eye as well. The Glaucoma leads to permanent blind conditions to the people and most of the people around the globe suffered from intraocular pressure (IOP) over an eye. This kind of IOP effect causes severe destructions in the optical nervous system which is connected towards the brain. The major objective of this work is to design a new framework for recognizing and predicting the Glaucoma disease with respect to digital image processing scheme. The authors specify that several methods were available in the past to identify the Glaucoma disease; however, the major problem identified with such implementations is time consuming in addition to irregular interval iterations. To avoid these issues, a new strategy with powerful classification norms is introduced in this paper with respect to Support Vector Classifier logic with multiple class specifications. This work is applied on a public dataset and respective dataset images gathered from eye clinic containing Glaucoma, no Glaucoma, and suspect for Glaucoma. This proposed strategy gives precision of 90% for fundus pictures and 92% for OCT retinal images and the recognizable proof of the glaucoma stage is accomplished by contrasting the aftereffects of both the retinal fundus picture and the OCT picture. The major advantage identified from this paper is the application of K-Means Optimization Logic to optimize the digital glaucoma images with high contrast to estimate the affected regions easily with respect to the proposed classification logic [16]. The image-based changes recognition is identified by using classical morphological procedures [17].

An et al. proposed a paper related to Glaucoma identification using machine learning principles with respect to OCT and fundus image analysis. In that paper, the authors illustrated that Glaucoma is a serious cause and it needs to be identified on initial stages via OCT scanning process. The application of machine learning technologies gives boost to the proposed approach and improvises the constraint of identifying such disease in a good manner. In this approach, around 208 image samples are considered for Glaucoma and 149 images are considered for normal eyes. The digital image processing strategy is applied to process these images and this approach highly concentrates on Open-Angle Glaucoma type. This approach utilizes the advantages of Convolutional Neural Network (CNN) and provides high range of efficiency in results. The 10-fold cross-validation technique had area under receiver operating characteristic curves of the Convolutional Neural Networks of 0.940 for shading fundus pictures, 0.942 for retinal nerve fiber layer thickness, 0.944 for macular ganglion cell complex thickness, 0.949 for plate retinal nerve fiber layer deviation maps, and 0.952 for macular ganglion cell complex deviation maps. The random forest logic consolidating the five separate Convolutional Neural Network models improved the 10-fold cross-validation AUCs to 0.963. So, the machine learning framework depicted accuracy ratios, in which it can precisely distinguish solid and Glaucoma subjects dependent on their removed pictures from OCT information and shading fundus pictures, and the framework should assist with improving the demonstrative precision in Glaucoma prediction [18]. The major advantage identified from this paper is the application of Convolutional Neural Network concept to process the OCT and fundus images with high precision values and accuracy levels are attained with proper logical ratios [19].

## 3. Proposed System Methodologies

The proposed methodology of this paper is designed based on the dual strategic machine learning principles such as Depth Optimized Machine Learning Strategy (DOMLS) and the Modified K-Means Optimization Logic (MkMOL). This paper introduced a new algorithm called DOMLS, which integrates all the digital image processing constraints together to provide an efficient solution to identify the Glaucoma disease on retinal area and reduce the time complexity ratio in a good manner. The proposed logic is useful to identify the glaucoma disease on earlier stages and provide proper predictions accordingly with respect to the machine learning strategies. This paper associates many techniques together to produce an efficient machine learning and optimization strategy called DOMLS with MkMOL. Based on the following digital image processing associations to identify the glaucoma disease are as follows: image acquisition and ROI selection, optical disc segmentation process, hue, saturation, lightness (HSV) plane enhancement, and optimization logic association and classification. All these details are illustrated in detail below.

### 3.1. Image Acquisition and ROI Selection

The input image acquisition process begins with the selection of OCT images from the image repository and the process of Region of Interest (ROI) selection depends on the accuracy factor. The ROI selection process allows the user to select the specific region of input to process further accordingly. The general formation of ROI selection is in rectangle format, in which it is easy to acquire only the selected part and apply the feature processing logic only to that extracted part for improving the accuracy levels in outcome.  Algorithm 1 illustrates the logic of image acquisition and ROI selection in a clear manner with proper Pseudocode specification.

### 3.2. Optical Disc Segmentation Process

Segmenting the optical disc is a significant and basic advance in making a casing of reference for diagnosing optical nervous system associations, for example, Glaucoma. In this manner, a solid optical disc segmentation procedure is vital for programmed screening of optical nervous system irregularities. The principle commitment of this paper is in introducing a novel optical disc division procedure dependent on integrating a sequential process on a restricted optical disc image. To keep the veins from meddling with the sequential cycle, to prevent the nerves from interfering with the continuous rotation, the painting technique is used. Also a significant commitment is to include the varieties in decisions among the ophthalmologists in distinguishing the optical disc limits and diagnosing the Glaucoma. The vast majority of the past summaries and investigations were prepared and tried dependent on just a single assessment, which can be thought to be one-sided for the ophthalmologist. Along with this, the proper disc segmentation leads to higher accuracy rates in result and provides the low iteration level in outcome.  Algorithm 2 illustrates the logic of optical disc segmentation process in a clear manner with proper Pseudocode specification.

### 3.3. Hue, Saturation, Lightness (HSV) Plane Enhancement

During the Red, Green, and Blue shading model alluded to the handling of color tones in the manual visual framework, the HSV color model is compared to the human impression of color likeness. In this paper, a projection of RGB vectors inside the RGB color space is figured out, in which it isolates nonchromatic from chromatic data. The projection is the manipulation which is equal to hue and saturation of the HSV shading space in the RGB color portion. It incorporates the psychovisual idea of human separation between colors of the HSV space into the physiological visual-based idea of the RGB space. With the perception of it, as opposed to the overarching assessment, it is conceivable to separate between colors dependent on human discernment in the straight math of the RGB color space. This opens additional opportunities in numerous fields of color image handling, particularly in the area of Glaucoma-based image processing schemes.  Algorithm 3 illustrates the model of HSV image plane enhancement feature construction process in detail with proper Pseudocode specifications.

### 3.4. Optimization Logic Association and Classification

The proposed methodology of DOMLS adapts the new methodology of optimizer called Modified K-Means Optimization Logic (MkMOL), in which the optimizer performs the cluster-based Glaucoma image optimization. The necessity of cluster-based approach is utilized in this paper because of attaining the higher accuracy ranges with lesser error ratio. The best optimum results will be gathered on each level of cluster considerations and the vector points of each set of Glaucoma images are considered as a global mean and process accordingly. The general K-means cluster logic is enhanced over this machine learning process and improves the accuracy by means of adding some new convolutions and shaping natures.  Algorithm 4 illustrates the logical flow of proposed optimization process and the scenario indicates the dual optimization flow with respect to HSV constructed images, so that the enhanced form of K-means logic is manipulated over this approach. The structured form of HSV images is reconstructed based on best possible cluster region outcome and the local optimization features of such process will improve the quality of prediction in results. These regions will be segmented by using segmentation principles. The classification model identifies those segmented Glaucoma image features, processes the matching optimized regions based on the training set, and produces the exact classification summary with respect to proposed approach of DOMLS.

## 4. Result and Discussions

In this description, the empirical analysis of the proposed approach Depth Optimized Machine Learning Strategy (DOMLS) is to be discussed in a detailed manner with practical proofs. This section proves the efficiency of analyzing the Glaucoma portion in the eye ball with respect to improved optimization strategies. The work is initiated with the selection of OCT dataset training phase with respect to image segmentation, dual convolution as mentioned in Algorithm 4, proposed MkMOL optimization, and the classification label marking. Once this process is completed, the testing process is initiated, and the input test image will be processed according to the same norms followed over the training phase. In this testing phase, single image needs to be processed and the output is produced according to the trained set of images and its classification labels. The entire process estimation clearly proves the performance ratio and measuring the Glaucoma portion exactly from the given input and produces the results accordingly with correct timeline estimations. The accuracy and performance measures of the proposed optimization approach MkMOL are estimated in terms of overall time taken to process with efficiency of Glaucoma disease identification on real-time clinical environments. The entire programming and analysis are composed by using specialized digital image processing tool called MATLAB and the resulting units are properly accumulated in a fine manner.  Table 1 illustrates the classical algorithm comparisons and the accuracy estimations with respect to Nerve Fiber Layer (NFL), Ganglion Cell Layer (GCL), Inner Plexiform Layer (IPL), Inner Nuclear Layer (INL), Outer Plexiform Layer (OPL), Optical Nerve Layer (ONL), and Retinal Pigment Epithelium (RPE).

**Figure 6:**
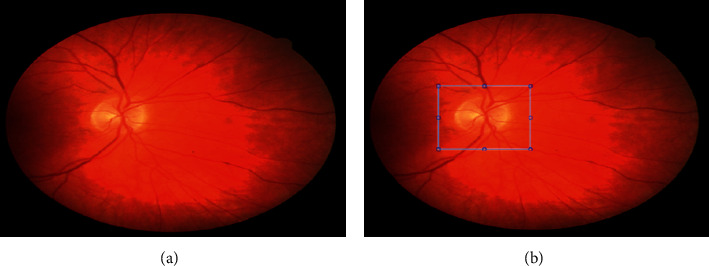
(a) Input image and (b) Region of Interest selection.

The following figures and their estimations prove the resulting accuracy of the proposed approach as well as the performance levels in detail. Additionally, in the outcome, the proposed approach of DOMLS proves the resulting accuracy level of 96.2%.  Figure 6 illustrates the input image acquisition and the Region of Interest selection process.

**Figure 7:**
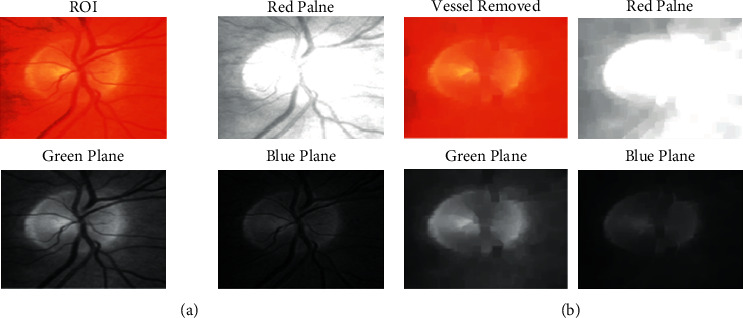
(a) Segmented ROI portion with proper HSV plane enhancement view and (b) Vessel Removed ROID and HSV plane enhancement view.

Figure 7 shows the view of extracted ROI portion from the input image and the optic disc view is more clear in comparson with Figure 6. The proposed optimization model Modified K-Means Optimization Logic based HSV plane enhancement model view is illustrated clearly in Figure 7(a), in which it shows the selected ROI portion of the image, as well as extracted Red, Green, and the Blue planes individually. The vessels are removed from Figure 7(a) and the actual plot is produced in Figure 7(b) in a visualized manner.

**Figure 8:**
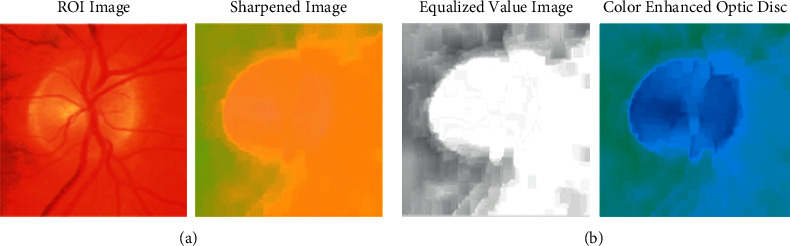
(a) Sharpened image. (b) Image equalization and color enhancement.

Figure 8 illustrates the sharpened image view model of the proposed approach in association with color enhancement portion of the sharpened portion of the Glaucoma image. The image equalizer logic is applied to the classical model of K-means and improvises the optimization logic in terms of accuracy enhancement to attain the best results in outcome and the resulting structure is shown below.

**Figure 9:**
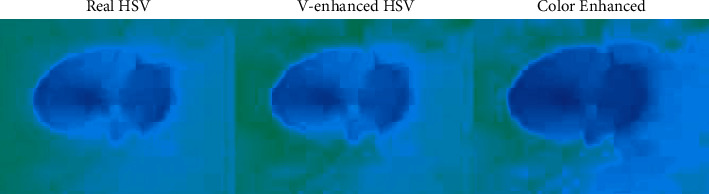
HSV based image enhancement model.

Figure 9 illustrates the HSV color conversion image view model of the proposed approach, in which it visualizes the hue, saturation, lightness (HSV) plane enhancement portion of the sharpened edges of the Glaucoma image. The following image shows the real portion of the input image and lighting enhanced portion of the image and the color enhanced portion of the input image in a clear manner.

**Figure 10:**
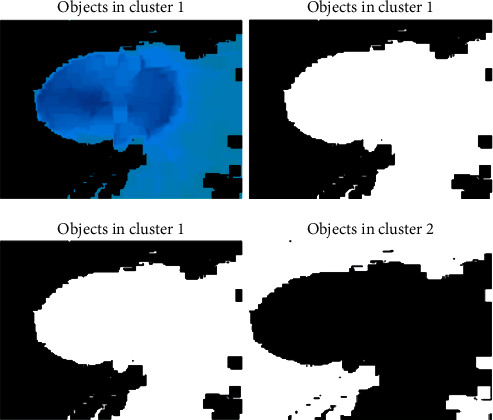
Cluster view with Glaucoma image objects.

Figure 10 illustrates the cluster-based image view model of the proposed approach, in which it visualizes the object presented into the created clusters and the model images, and the object representations are clearly shown below.

**Figure 11:**
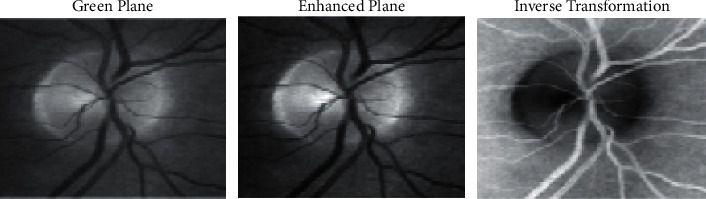
Cluster view with Glaucoma image objects.

Figure 11 illustrates the plane image view of different proportions including vector transformation as well, in which it visualizes the green plane view, enhanced plane view, and the inverse transformation view of the proposed approach. The transformation logic is inspired from the classical formulation specified in base transformed algorithm.

**Figure 12:**
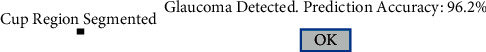
Cup region segmentation with resulting accuracy prediction.

Figure 12 illustrates the cup disc segmented region of the proposed approach and the resulting alert box initiations with detection alert as well as accuracy indications of the proposed optimization and machine learning model.

**Figure 13:**
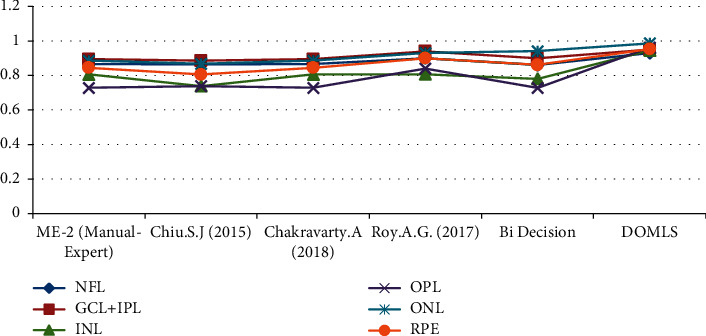
Analysis of public dataset retina-layer coefficients with respect to past research summaries.

Figure 13 illustrates the graphical perspective of the proposed approach accuracy level estimations with respect to the comparisons of several classical literature retinal features. These graphical estimations are acquired from different past research summaries, and the summarization view is clearly specified in the mentioned table, Table 1.

## 5. Conclusion and Future Scope

In this paper, an advanced machine learning algorithm called Depth Optimized Machine Learning Strategy (DOMLS) based Glaucoma disease detection strategy is implemented and the result is attained successfully; and all resulting scenarios are analyzed properly with experimental proofs. The proposed system simulation is implemented using machine learning strategy on the MATLAB tool and the highest accuracy range of around 96.2% is attained, as well as the resulting section figure; Figure 12 shows the proof for that. The segmentation portion clearly extracts the affected region with the help of Region of Interest selection procedures and the resulting figure; Figures 6 and 7 illustrate that with practical proof. The color saturation results are also perfect, as well as the resulting figure; Figures 8 and 11 illustrate that with practical proof outcome scenario. This paper utilizes the advantages of new optimization logic, in which the implementation model algorithm is constructed newly and the mentioning is given clearly in Algorithm 4. The feature extraction and classification process over the proposed approach had good accuracy in outcome. The proposed approach of DOMLS with MkMOL proves the efficiency of the Glaucoma disease detection problem in a clear manner, and the resulting section clearly illustrates the process of segmentation and classification process. For all the proposed results clearly demonstrate the efficiency of the proposed algorithm and it is more efficient and fine in working with accuracy levels of 96.2%

In the future, the work can be extended or revised by means of adding the deep learning principles on training phase with respect to the identification of all kinds of dataset to predict any category of Glaucoma disease in an efficient manner and include some more time related features to the newly proposed optimization scheme called Modified K-Means Optimization Logic to attain good accuracy levels of around 98%.

## Figures and Tables

**Algorithm 1:**
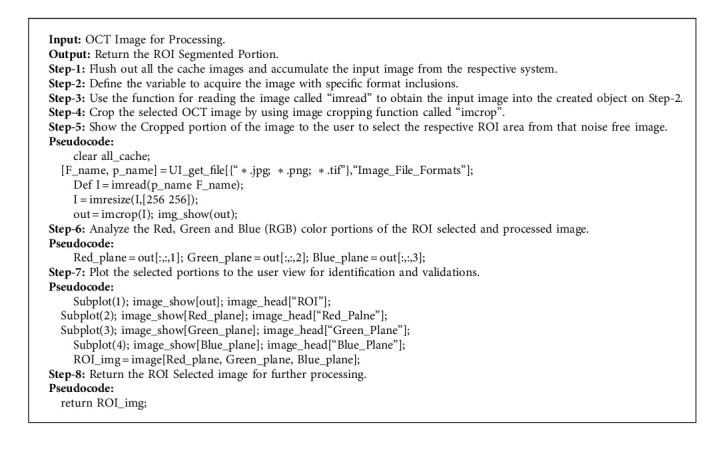
Image acquisition and ROI selection

**Algorithm 2:**
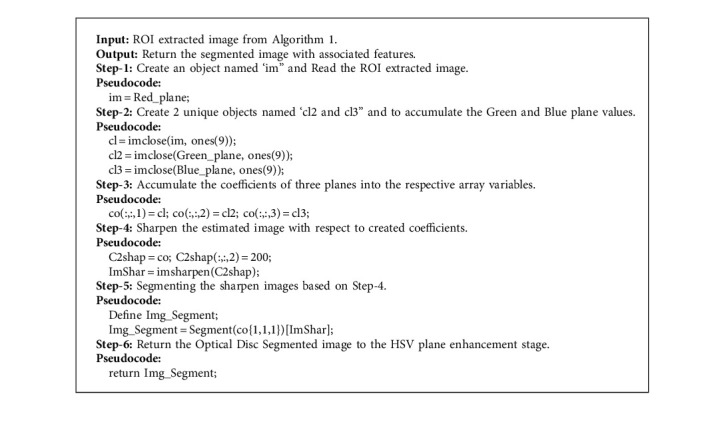
Optical disc segmentation process

**Algorithm 3:**
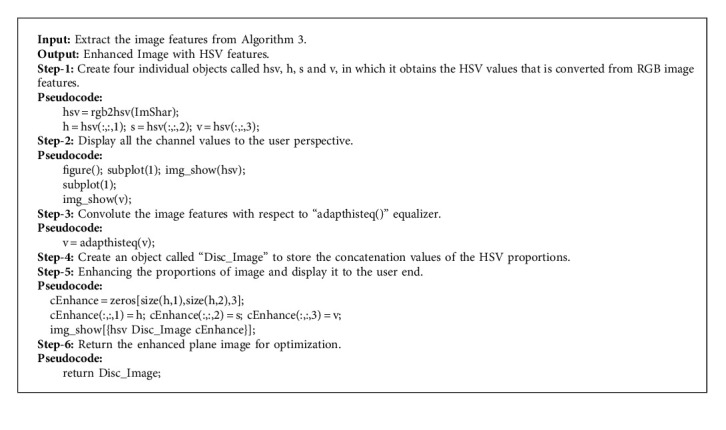
HSV plane enhancement

**Algorithm 4:**
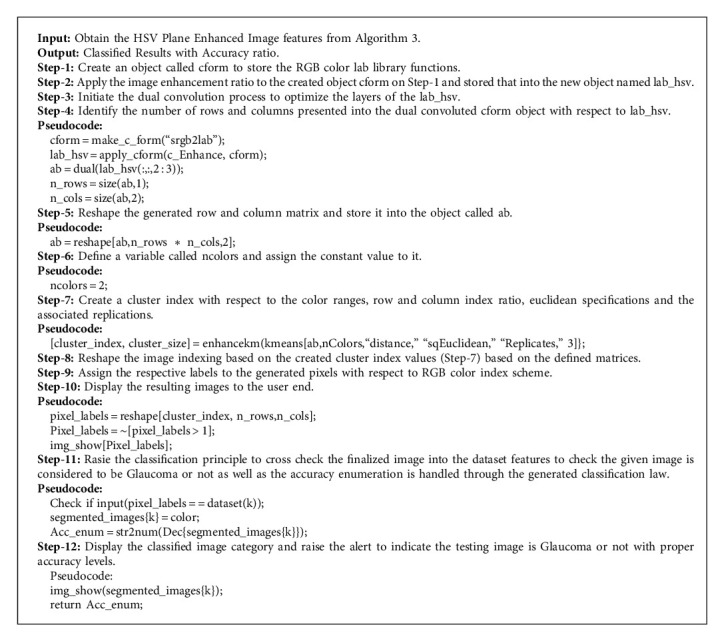
Optimization and classification

**Table 1:** Comparison view of public dataset retina-layer coefficients. The proposed approach's view is in bold.

	NFL	GCL and IPL	INL	OPL	ONL	RPE

ME-2 (Manual-Expert)	0.867	0.895	0.806	0.729	0.886	0.845
Chiu [9]	0.865	0.886	0.738	0.739	0.868	0.806
Chakravarty [20]	0.867	0.895	0.806	0.729	0.886	0.845
Roy [21]	0.90	0.94	0.87	0.84	0.93	0.90
Bidecision	0.861	0.900	0.781	0.728	0.941	0.861
DOMLS	**0.93**	**0.95**	**0.946**	**0.962**	**0.986**	**0.953**

## Data Availability

The data used to support the findings of this study are included within the article.

## References

[1] Aljazaeri M., Bazi Y., AlMubarak H., Alajlan N. Faster R-CNN and DenseNet regression for glaucoma detection in retinal fundus images.

[2] Govindaraj D., Logashanmugam E (2018). Study on impulsive assessment of chronic pain correlated expressions in facial images. *Biomedical Research*.

[3] Saxena A., Vyas A., Parashar L., Singh U. A glaucoma detection using convolutional neural Network.

[4] Borwankar S., Sen R., Kakani B. Improved glaucoma diagnosis using deep learning.

[5] Shifani S. A., Megalan Leo L., Yokesh V., Karthikeyan K. V., Ramkumar G. (2019). Skin lesion detection based on fuzzy logic. *International Journal of Innovative Technology and Exploring Engineering*.

[6] Ramkumar G., Logashanmugam E. An effectual face tracking based on transformed algorithm using composite mask.

[7] Kanakatte A., Gubbi J., Ghose A., Purushothaman B. A decision support system for retinal image defect detection.

[8] Amirthalakshmi T. M., Ramkumar G., Vijayakumari P., Samuthirapandi V. Effective control of non linear parameters using artificial intelligence.

[9] Chiu S. J., Allingham M. J., Mettu P. S., Cousins S. W., Izatt J. A., Farsiu S. (2015). Kernel regression based segmentation of optical coherence tomography images with diabetic macular edema. *Biomedical Optics Express*.

[10] Nugraha G. S., Riyandari B. A., Sutoyo E. RGB channel analysis for glaucoma detection in retinal fundus image.

[11] Karkuzhali S., Mishra A., Ajay M. S., Wilson Prakash S. Glaucoma diagnosis based on both hidden features and domain knowledge through deep learning models.

[12] Palakvangsa-Na-Ayudhya S., Sapthamrong T., Sunthornwutthikrai K., Sakiyalak D. GlaucoVIZ: assisting system for early glaucoma detection using mask R-CNN.

[13] Bedke G. C., Jadhav M. E., Punde P., Dongaonkar S. Retinal OCT images for glaucoma.

[14] Govindaraj D., Logashanmugam E. (2019). Multimodal verge for scale and pose variant real time face tracking and recognition. *Indonesian Journal of Electrical Engineering and Computer Science*.

[15] Wang J., Wang Z., Li F. (2019). Joint retina segmentation and classification for early glaucoma diagnosis. *Biomedical Optics Express*.

[16] Fathima C. S., Subhija E. N. Glaucoma detection using fundus images and OCT images.

[17] Agnes Shifani S., Ramkumar G., Nanammal V., Thandaiah Prabu R. (2020). Exploration of morphological procedure on the recognition of fundus image. *Journal of Computational and Theoretical Nanoscience*.

[18] An G., Omodaka K., Hashimoto K. (2019). Glaucoma diagnosis with machine learning based on optical coherence tomography and color fundus images. *Hindawi Journal of Healthcare Engineering*.

[19] Ramkumar G., Logashanmugam E. (2018). Hybrid framework for detection of human face based on haar-like feature. *International Journal of Engineering & Technology*.

[20] Chakravarty A., Sivaswamy J. (2018). A supervised joint multi-layer segmentation framework for retinal optical coherence tomography images using conditional random field. *Computer Methods and Programs in Biomedicine*.

[21] Roy A. G., Conjeti S., Karri S. P. K. (2017). Relaynet: retinal layer and fluid segmentation of macular optical coherence tomography using fully convolutional networks. *Biomedical Optics Express*.

